# Promoting medical student engagement through co-development and peer-assisted learning: a new patient safety course as a case study

**DOI:** 10.1186/s41077-022-00212-5

**Published:** 2022-06-06

**Authors:** Jesper Dybdal Kayser, Anne Mielke-Christensen, Doris Østergaard, Peter Dieckmann

**Affiliations:** 1 Copenhagen Academy for Medical Education and Simulation (CAMES), Herlev Hospital, Borgmester Ib Juuls Vej 1, Entrance 1, 25th floor, DK-2730 Copenhagen, Herlev Denmark; 2 Faculty of Health and Medical Sciences, University Copenhagen, Copenhagen, Denmark; 3 Faculty of Health Sciences, University Stavanger, Stavanger, Norway; 4 Department of Clinical Medicine, University of Copenhagen, Copenhagen, Denmark

**Keywords:** Peer-assisted learning, Near-peer teaching, Curriculum development, Student facilitator, Medical students, Patient safety, Non-technical skills, Simulation

## Abstract

**Introduction:**

Peer-assisted learning programs have been focused on providing students with competencies to deliver lectures and facilitate workshops, whereas involvement of students as co-developers of educational programmes has been relatively under-described in the literature. Likewise, the use of students as facilitators in simulation-based training and debriefing is also scarce.

In this paper, we describe how medical students were co-developers of a novel course on patient safety and how they were trained as student facilitators to conduct simulation-based training and debriefing, as well as workshops.

**Methods:**

Medical students co-developed a course in patient safety consisting of three simulation-based scenarios and three workshops. The students were educated in relevant patient safety topics. They were trained to become student facilitators to conduct workshops, simulations and debriefings at a patient safety course for medical students. A questionnaire was developed to evaluate the course participants´ perception of the learning objectives and the student facilitators following the latest course in 2020. In addition, semi-structured interviews with the student facilitators were conducted to explore their perceptions of being part of the course.

**Results:**

A total of 92% of the course participants completed the evaluation of the course. The majority of the course participants found that the student facilitators created a safe learning environment and had the necessary skills to teach. The learning objectives for the course were found to be useful. A total of 10 interviews with the student facilitators were conducted. We found that the student facilitators were motivated to teach in the course, as a way of improving their teamwork, leadership qualities and communication skills, as well as their resume. Some of the student facilitators mentioned that they were able to create a safe learning environment, whereas others mentioned a feeling of inadequacy for their teacher role. In addition to developing their teaching skills, they mentioned that they developed their medical expertise, alongside their communication-, collaboration-, leadership- and professional skills.

**Conclusion:**

This study illustrates how medical students were involved in the co-development, delivery and implementation of a course in patient safety. The evaluation of the course shows that student facilitators succeeded in creating a safe learning environment. The interviews of the student facilitators reveal their various motivations for teaching, in addition to different perceptions of their experience as a student facilitator. Some expressed a positive feeling of being able to establish a safe learning environment, whilst others expressed a feeling of inadequacy when facilitating peers. In addition, the student facilitators indicated that they developed themselves both professionally and personally.

## Introduction

In recent years, there has been a push for student engagement within undergraduate medical education. This call for student involvement, however, has not been met by specific examples illustrating how educators may concretely engage students in their institutions [1, 2]. Two distinct areas of students¨ engagement that have been relatively under-described in the literature are students as co-developers of curricula and students as peer teachers.

The Aspire-to-Excellence initiative has provided a comprehensive framework on student engagement activities and involvement in curriculum development is specifically mentioned as a key domain [1, 3]. The reported activities range from medical students gathering feedback to a role as module co-director [4].

Peer-assisted learning has gained prominence through communities of practice as a theoretical lens, which highlights the role of purposeful interaction and joint enterprise amongst community members, to promote skill development [5]. It has a positive impact on learners [6, 7]. In problem-based learning courses and clinical skills instruction, medical students’ performance on tests of knowledge or skills is similar, irrespective of whether they were taught by faculty instructors or peer teachers [7–9]. However, the evidence for the usefulness of near-peer teaching have focused on the practical gain for student learners, and therefore, less is known about the gain for the peer-teachers [10]. This perspective is important as the peer-teachers might benefit in their learning from teaching others.

Peer-assisted learning programs have been focusing on providing the students with the necessary competencies to deliver lectures and facilitate workshops [11]. However, studies evaluating near-peer teaching for more complex topics such as patient safety are scarce [12]. Likewise, studies of students as facilitators in simulation-based training (SBT) and in debriefing are also scarce. A recent study describes the feasibility of involving medical students in simulation-based scenarios and debriefing of their peers [13].

Enhanced understanding about how to meaningfully engage students in these processes as co-developers for a new curriculum, as well as the related learning resources, could be a valuable next step to improve the current state of the art of peer-assisted learning in health care. In addition, students should be incorporated in the faculty team, as peer-teachers, for the course. This will help undergraduate medical education curriculum designers to develop courses that better meet the needs of their students.

In order to describe and assess the impact of student engagement in undergraduate medical education on both student learners and student facilitators (SFs), we actively involved medical students in the course development and delivery of a new simulation-based patient safety curriculum. We used a novel patient safety course, due to the global importance of this topic, whilst simulation-based training was chosen as the teaching method, given its increased complexity when compared to conducting a lecture or workshop.

### Objectives

In this paper, we describe how medical students were co-developers of a novel course on patient safety and were trained as SFs to conduct SBT and debriefing. In addition, we describe the course participants´ reactions and learning points from the course and the medical students’ experiences as SFs.

## Methods

The study is a mixed-methods study. Quantitative data was obtained using a questionnaire to evaluate the course participants reaction. Qualitative data were obtained by individual interview of the SF.

### Context

The Copenhagen University is the largest university in Denmark, with a yearly uptake of around 600 medical students. The duration of the undergraduate medical program is six years. The patient safety curriculum is integrated into the 4^th^ year. The curriculum includes lectures and workshops conducted at the university campus and a 1-day mandatory course conducted at the regional simulation centre, the Copenhagen Academy for Medical Education and Simulation (CAMES). Co-development refers to the collaboration between SFs and faculty.

### Course development method

Course development was based on Kern’s six steps for curriculum development [8, 14] An overview of each step, followed by the actions taken are shown in Table 1. The development team consisted of four medical students and two faculty members (DOE and PD).

**Table 1:** Kern’s six step approach for curriculum development, background and actions taken

Kern’s six step approach for curriculum development	Background	Actions taken
Problem identification and general needs assessment	• Patient safety concerns is a serious and global health care problem • Future health care professionals need knowledge and skills to make them able to predict and deal with the risks surrounding a complex healthcare society • AMEE recommends that patient safety education should be integrated in the education of undergraduate medical students	• The University decides to develop a patient safety curriculum • A 1-day mandatory course is to be developed and conducted in the regional simulation center
Targeted needs assessment	• WHO provides a comprehensive curriculum guide for medical schools	• We adapted the curriculum to local context based on discussions with patient safety experts and focus group interviews with a total of 20 medical students. • The interview guide covered o What is patient safety? o Learning from errors to prevent harm o Understanding and managing clinical risks • The transcripts were analyzed and the most important subjects for patient safety were leadership, communication and teamwork
Goals and objectives	• The overall goal was to bring medical students´ technical and non-technical skills to a level thar could increase patient safety	• The learning objectives were grounded in the WHO curriculum and the needs analysis. The content of the course is seen in Table 2.
Educational strategies	• Pre- course material • Interactive learning methods	• Online video presentations • Workshops • Role playing • Simulation-based training and debriefing
Implementation	• The course is mandatory • It is implemented in the 4th year at the beginning of the medical students´ internal medicine/surgical internship	• A total of 58 courses have been conducted from 2016 to 2020 • The number of course participants are 2,226
Evaluation and feedback	• Evaluation of the course is part of the evaluation of the full patient safety curriculum	• Verbal evaluation and questionnaire at the end of the day • Interview with peer teachers

### Involving medical students as co-developers

The SFs involved in the development of the Patient Safety Course were selected from a group of students already employed by CAMES. From the pool of 40 medical students, applicants were invited to sent a motivated application to the faculty by email. A handful of those applicants were invited to a job interview. New SFs were chosen based on their motivational factors like education and course development, as these two are the main tasks of the SF. The SFs were also chosen based on their ability to think and work independently as we believe that these abilities are important.

The students fulfilled several roles at CAMES [15], including controlling the high fidelity simulators and acting as simulated patients in scenarios. This implied that they already had an understanding of SBT as well as the technical and non-technical skills involved in this approach.

We set out to involve SFs throughout this process, from the initial development of the course, to its delivery and continuous revision. Our assumption was that by involving SFs in the course development process, the resulting content would be more relevant for course participants. The SFs were also part of the target group for the course and thus, could help in identifying challenges typically experienced.

The SFs were involved in preparing the objectives for the course, preparing pre-course material, including relevant literature and short online video presentations as preparation for the course. A combination of interactive workshops and simulation-based scenarios were also developed, with the scenarios being followed by debriefing. The SFs drafted a first version of the material after an initial discussion in the team, and then iteratively revised the material until agreement was reached. The course was intended to provide a wide range of learning opportunities and allow the participants to train in a safe learning environment. A complete overview of the course is seen in Table 2. The length of a scenario and debriefing session was 45 min.

**Table 2:** Overview of the content and methods used in the Patient Safety Course

Time	Content	Method
08:00-08:25	Introduction to faculty Key concepts of patient safety	Lecture
08:30-09:20	Speak-up Barriers of speak-up in the clinical setting	Workshop Group-discussion
	Coffee break	
09:25-10:00	Pro´s and con´s of being new in a department Team dynamics´ influence on working processes	Workshop Role-play
10:10-11:35	Human factors’ influence on the use of medical equipment and devices	Workshop Group-discussion
	Lunch break	
12:25-13:20	The use of the ABCDE approach Situational awareness in the context of ABCDE Teamwork in the context of ABCDE	Simulation and debriefing
	Coffee break	
13:30-14:25	SBAR communication technique Insight in your own competences and weaknesses When to offer help and when to say no as it exceeds your abilities	Simulation and debriefing
14:25-15:20	Informing a patient about a medical error Patients’ and healthcare professionals’ responsibilities and rights Importance of checking patient identification Insight on how errors occur and how we can minimize them Insight in taking care of one’s self – the “second victim”	Simulation and debriefing

The course was designed to accommodate cohorts of 40 medical students. The group was divided into two – a simulation group and a workshop group, of 20 participants each. The simulation group was further divided into three groups of 6-7 participants. All medical students participated in both the workshops and SBT. To deliver the course, at least five SFs were necessary, one for each of the three simulations and two for the workshops. In addition to the SFs, at least one junior doctor was present throughout the course in case questions requiring clinical experience arose. At times, this person emphasized the relevance of the topics addressed in actual care as not all students immediately recognize the relevance. Additionally, one of the faculty members delivered the morning lecture on patient safety, whilst an experienced nurse practitioner acted as a co-teacher for the human factors´ session.

### Training of the NPTs

Initially a total of 10 SFs received both formalized instructions and reviewed literature on how to be a facilitator in workshops and SBT scenarios, with a particular focus on debriefings, as this was a new field for all of them. The simulation-debriefings were structured in three phases: description, analysis and application [16]. A course manual was produced by the SFs, and subsequently reviewed by faculty, to standardize the courses as much as possible and assist new SFs in delivering feedback and debriefings. The SFs were supervised by the two faculty members during the first four courses. After the first team of SFs had been trained by faculty, new SFs were trained by experienced SFs and the faculty. Firstly, they observed three different SFs conducting the simulation scenarios, and then they lead the debriefing themselves under supervision. After the SFs had practiced for some months, they participated in a three-day standardized instructor course at CAMES, that aims to train them as independent facilitators [17]. For the first six months, newly employed SFs conducted the simulations only. After six months, they were also supervised in running the workshops. The reason for this was that the workshops were considered more demanding in terms of facilitating the discussion. The SFs regularly supervised each other, and occasionally, they were supervised by faculty to further develop their competence.

For the current course, we anticipated several benefits of the co-development process and the SF concept with supervision by faculty. Firstly, the SFs likely understand the challenges and viewpoints of the learner, as their respective experiences are similar. Secondly, the fruitful discussions in the co-development process and the supervision of the course implementation by faculty will allow for fine-tuning the content of the delivery. Finally, it may help the SFs recognize the limits of the discussions, e.g. if the learners had questions outside of the learning objectives.

### Data sampling

A questionnaire was developed to obtain the students evaluation of the learning objectives, the learning environment, the competences of the SFs and the effect of near-peer teaching. The questionnaire was developed through discussion and piloted for understanding by 10 course participants on a Patient Safety Course. Thereafter, each question was discussed within the team and changes were made accordingly. The final version of the questionnaire consisted of 21 questions. A five-point Likert-scale design, in which 5 indicated the best rating, was adopted. The questionnaire was administered to participants at the end of the course in February 2020. In addition, the course participants’ verbal feedback was collected and transcribed by the NPT after each course and comments from the NPT were collected. Once every semester, these evaluations were discussed between NPT and faculty and the course was adjusted accordingly.

To evaluate the long-term effect of being an NPT, semi-structured interviews with NPTs were conducted by JK. An interview guide was developed by the study team, pilot tested by JK and revised accordingly. A convenience sample of 10 NPTs were invited to participate (all the first NPT trained). The interviews were conducted over telephone and audio recorded. The interviews lasted between 25 and 28 minutes. They were fully transcribed by JK and read by all authors.

### Data analysis

The data was analysed using the six-step thematic analysis approach, described by Braun and Clarke 2006 and Kiger ME 2020 [18, 19]. After familiarization with the data, initial codes were generated by JK. The codes were discussed, and themes identified. The themes were then clustered, based on similarity in meaning. Once again, the clustered themes were discussed by all authors and changes were made accordingly. After the authors had agreed on the clustered themes, JK broke the themes down into subthemes to ease the understanding of what the clustered theme consists of. The subthemes were then discussed in the team and changes were made accordingly.

## Results

A patient safety course focusing on human factors, leadership, collaboration and communication was co-developed by faculty and NPTs. A combination of interactive workshops and simulation-based scenarios, followed by debriefings, were used as educational methods.

### Course participants evaluation of the course and the NPT

A total of 71 course participants completed the questionnaire following the two separate course days in 2020. Six course participants out of 77 did not wish to participate. This resulted in a response rate of 92%. Table 3 shows the course participants` evaluation of the course and the SFs. The majority agreed or strongly agreed that the patient safety curriculum was useful for them and that the SFs had the necessary skills to teach this course.

**Table 3:** The course participants’ evaluation of the course and of the student facilitators *N* = Number of respondents

	N	Strongly disagree %	Disagree %	Neutral %	Agree %	Strongly agree %
**When I become a doctor the curriculum on … will be useful**						
*speak-up*	71	1	1	6	38	54
*reflecting on barriers regarding speak-up*	71	3	0	13	35	49
*being new*	71	1	1	14	24	59
*team dynamics*	69	1	1	10	43	43
*how human factors influence on handling medical equipment, and how adverse events can happen*	71	3	0	6	42	49
*being motivated to seek out knowledge on medical equipment*	70	1	1	10	47	40
*the use of the ABCDE-approach*	71	1	0	0	10	89
*situational awareness*	70	1	0	11	31	56
*teamwork*	71	1	1	4	32	61
*the ISBAR-tool*	70	1	0	3	22	73
*knowing your own strengths and limitations*	71	1	3	0	34	62
*saying no, when asked something that is beyond your competences, and knowing when to offer your help*	71	1	1	11	31	55
*informing patients of adverse events, such as being given the wrong medicine*	70	1	1	0	21	76
*the patient and health care professionals’ responsibility and rights regarding adverse events*	71	1	1	3	41	54
*checking patient identity*	71	1	1	6	34	58
*how to talk to patients about adverse events*	71	1	0	1	31	66
*knowing how and why medication errors occur, and how the amount can be minimized*	71	1	1	7	38	52
*second victim*	71	0	4	13	37	47
**I thought the …**						
young doctors had the necessary skills to teach this course	71	1	0	0	14	85
medical students had the necessary skills to teach this course	71	1	0	1	24	73
young doctors created a safe learning environment	71	1	0	1	14	83
medical students created a safe learning environment	71	1	0	0	16	83

### Semi-structured interview of the peer teachers

Table 4 shows the result of the analysis of the 10 interviews with SFs. The themes and sub themes are presented together with the citation which most effectively outlines the meaning of the theme.

**Table 4:** Themes, sub themes and selection of citations from the interviews of the near-peer teachers

Themes	Sub themes	Citation
**Motivation for being a teacher**		
**Intrinsic factors**	Improvement of non-technical skills	*“I really wanted to work with teamwork, leadership, and communication.”*
	Making a difference for the student	*“Well, it’s probably about seeing (…) that you are making a difference for the students and their education.”*
**Extrinsic factors**	Improvement of resume	“*This was also an opportunity to improve your CV and career as much as possible before you graduated.”*
	A future career in education	“*But it was also about finding it extremely interesting and hoping to continue in medical education alongside your regular job after graduating.”*
**Near-peers’ perceptions of being a co-developer and a teacher**		
**My role as a co-developer**	Involvement	*“I feel that we are given quite a big degree of freedom in developing the course.”* *“It works so well! It is us that makes it. Us that are actually there and knows what is going on. So we need to be part of it. In collaboration with the people higher up, so we know what resources are available. And it is a bit different in comparison to the other courses. It is so great, but it is something that you need to know. You need to know that it is expected of us to help develop the course this much. It is such a cool idea.”*
**My role as a teacher**	Take responsibility	*“Well, as a teacher it requires so much more responsibility. You are responsible for everyone being able to keep up. All the students need to play an active part and learn something. It is also your responsibility to help the shy and more restrained students to be active. Activating people. Responsibility for keeping the time and sticking to the structure.”*
	Maintain overview	“*You need to be more observant and reflective when watching the simulation (…) It is like being a conductor. The broad perspective is everything.”*
	Establish a safe learning environment.	*“It is one of the strengths of the patient safety course, that we create a safe learning environment. Maybe easier as we are at the same place in the hierarchy.* *It makes reflection possible and enables us in discussing communication on a more emotional level, where all ideas and thoughts are permitted. This is seen in contrast to some senior doctor who just say: “This is the way things are, deal with it””* *The students do not perceive you as an assessor*
	Enhances the reflections made by both the student and the teacher	*“Your insight as a medical student is clearer. Your teaching is more structured and focused on how other medical students think. Because you’ve been there yourself. I also think the students find the teaching more relatable, and perhaps they’ll learn more from this class when it is relatable. You’re on the same level, and not very far from each other regarding hierarchy.”*
**Challenges**	Unexpected things happening during sessions	*“That thing about learning to understand what other people are thinking. Why they make the decisions and choices they do, and then trying to evaluate and reflect on that in the group. It also helps you process your own ideas on how things are and should be and are connected. I find that extremely challenging as it can undermine your own perception of life.”*
	Knowledge questioned	*“Well, I’ve always had the notion that the teacher should be the one who enlightened the students, the one who teaches and makes the students smarter. When your knowledge gets questioned, it can be difficult to be a reliable source on the subject you are trying to teach.”*
**Facilitators**	Previous experience as a teacher Feedback	*“It helped the transition. I think if I hadn’t had the opportunity (to teach other places) then it would have been a much harder transition to teaching the patient safety course. The learning curve has been very steep.”* “*I received great feedback from the three teachers that helped train me, they also had some great tips and points. Great thoughts in general on the subject we teach. Also, there were some* *teachers that sent me material I could read up on beforehand. Read it to improve yourself. And I do expect this to be even more pronounced working with medical students and doctors.”*
	The position as teacher grants respect	*“You also get a lot of respect just from being their teacher. People look at you and think: Oh, you’re the teacher. And that makes them respect and listen to you automatically. At least that’s my experience.”*
**Near -peer-teachers self-development according to the seven roles of the medical doctor**		
**Medical** **expert**	Knowledge and skills	*“I reflect on all the things that we teach the students every time I come home. I’ve improved my knowledge on the subjects we teach and have become much more attentive to speak-ups for example. In a perfect world, I would be a lot better at speaking up, and I still feel that there are many situations where I don’t.”* *To take the medical knowledge and skills with you into clinical practice*
**Communicator**	It is more than listening Can be used in other situations	*“I think more about communication and how important proper communication is to our clinical work. Being able to hear the subtext of what people are saying, and not just listening to their words.”*
**Collaborator**	Collaboration with the nurses Collaboration with peers	*“Yes, it has been really great working with the experienced nurses. They are much more in touch with their technical skills. They know how it is in real life. Because medical students don’t really have this practical and technical experience. The nurses can provide a more clinically focused discussion (…) I actually feel that we couldn’t really vouch for our course without them. Because they actually know how things are.”* *“We helped each other with content, good ideas of how to do it”*
**Leader**	The role of the facilitator	*“The role as a facilitator is like being a conductor, it is the broad lines – having an overview and leading the team in the right direction”*
**Professional**	Being a professional	*“To stand in front of a group of people telling them something and make them listen (…) It somehow requires interprofessional competencies”* *“I would like to become a very good doctor and the seven roles of a doctor defines very well what a good doctor is”*
**Scholar**	Complexity of teaching	*“Standing in front of a group and understanding what their needs are and catching the balls that they throw up in the air. Facilitating a structured discussion and helping them to reflect on the things that matter to them. Really listening to the students and hearing the non-uttered elements.”* *“In general, I wish to develop as a teacher, and this I feel the course leaders have contributed to.”* *“I don’t really think I knew how complex it is to teach. This is what made the instructor course so good. It opened your eyes to the complexity of teaching. It gave you some tools to feel more at ease.”*

The SFs were motivated to teach in the course by the idea of improving their own ability to teamwork, leadership and communication skills, as well as their resume. Making a difference for the medical student was also mentioned. The SF’s perception of peer-to-peer teaching included reflections on their responsibilities and competences. Some SFs had a feeling of inadequacy when teaching, whereas others felt that near-peer teaching created a safe learning environment that inspired reflection in both medical students and them-selves. The SFs also talked about a feeling of increased responsibility when working as an SF. In addition, they felt they were developing their professional and medical expertise. SFs mentioned that the faculty members created an environment in which they could develop as a SF.

## Discussion

### Main findings

Medical students were successfully involved as co-developers and SFs in the delivering of workshops and SBT in a 1-day mandatory simulation-based patient safety course. The course participants expressed that the SFs had the necessary knowledge to deliver the content of the course. The interviews with SFs showed their different motivations for teaching. In addition, that they were developing themselves through teaching. Overall, the concept of co-developing and near-peer teaching were well suited for the topic of patient safety.

### Co-development of the Patient Safety Course

We opted to involve SFs in the development of the Patient Safety Course and the delivery of SBT and workshops. In particular, by giving them responsibility for the course material, they became real working members of the team.

We chose to involve SFs even though patient safety is a new field for them. The SFs understand the learning needs of their peers and know which challenges the course participants face in the clinical setting [15]. A recent study has shown that the curriculum benefits from the students´ involvement in its development, as it includes the students´ perspective and aids its continuous improvement [4]. A long site that, Nunnink et al found it feasible to involve senior medical students in the development of SBT scenarios [13]. Involving the stakeholders from the beginning is in agreement with one of the 12 tips for implementation of “student as teacher” programs [11] and global standards for quality improvement [17].

Other universities have successfully implemented patient safety courses for medical students containing the same elements of WHO’s curriculum guide for patient safety [20–22]. However, we chose to use a combination of interactive learning methods and involving SFs.

### Medical students as SFs

One of the main reasons for using near-to-peer teaching was to target the appropriate level of learning in a safe learning environment, Secondly, this approach keeps costs low and allows efficiency in course development, whilst still delivering high quality content. We found that the course participants’ reaction to the perceived skills of SFs was positive, which is in accordance with other studies of the field [7–9, 23]. We did not evaluate the learners´ outcome in this study, but according to the literature, peer-assisted learning enhances student learning [11]. In addition, a systematic review has previously shown that peer-assisted learning achieves learner outcomes that are comparable with those produced by faculty-based teaching [10]. Finally, the SFs have the chance to improve their own ability to teach with different methods.

Our approach was new in using the SFs in workshops, SBT and debriefings, where the topic was also new for them. This is in agreement with a recent study showing that peer-assisted learning in SBT is feasible (NUNNINK). We focused on developing a formal training program for the SFs. This covered the relevant aspects of patient safety and human factors, providing feedback to course participants and facilitating the debriefing after simulation-based scenarios. The SFs were supervised by faculty, both initially and during the maintenance phase. Faculty aimed to promote a longitudinal relationship with the SFs to stimulate their continuous development and their ability to train new SFs. The SFs were also invited to faculty development meetings to discuss further developments. This is in agreement with the recommendations for students as teachers programs [11] and in line with the literature about students as partners with staff [24].

All of the SFs became involved whilst they were medical students, and they can continue for two years after graduation and use their newly collected experiences as junior doctors to give relevant inputs to the patient safety course. As previously mentioned, we recruit SFs from our pool of 40 medical students employed by CAMES, to assist with running SBTWe opted to pay the NPTs, as we already pay the students for running the simulations. The salary is based on an agreement with the students’ union. This is in contrast to other universities, which advocate for this to be a voluntary experience for the NPTs [5].

### The SF’s motivation

The interviews showed that the SFs had different motivations for teaching. The motivations can be categorized as either intrinsic motivation, which refers to doing an activity for the satisfaction of the activity itself or extrinsic motivations, which refers to the performance of an act to attain a separable outcome [25, 26]. SFs were motivated by the possibility to improve their non-technical skills and by hoping to make a difference for the students and their education. These can be categorized as intrinsic motivators, which have been shown to enhance performance, persistence and creativity [25, 27]. Intrinsic motivations can be the tendency to seek challenges, to extend and exercise one's capacities, to explore, and to learn [28]. In our study, extrinsic motivation were external rewards such as an improved resume with the motivation of enhanced career-opportunities. The peer teacher, who is motivated by a better resume is therefore, extrinsically motivated. Our findings of extrinsic and intrinsic motivations for teaching at the patient safety course are in accordance with other studies of teaching [29, 30].

### The SF’s perceptions of their experience

The SFs had different perceptions of peer-to-peer teaching. One aspect is a positive reflection of being able to establish a safe learning environment, which inspired reflections in both peer teachers and learners. The second, contrasting aspect is a feeling of inadequacy, due to having the same position in the hierarchical system and not necessarily possessing much more knowledge on the subject than their peers. This could have been exacerbated by the relative inexperience in delivering simulation-based teaching. This is in agreement with findings, describing the experiences of more senior staff, who are new simulation facilitators [31, 32]. Another reason for the SF’s feelings of inadequacy could have been the ‘fear of being wrong’. In the training program, we emphasized that it was acceptable to answer: “I don´t know”, if they did not know the answer to a question and that this could be addressed in a later plenum session. This is in agreement with the recommendations by Freret et al. [11]. Several studies of peer-to-peer teaching support the findings that they can have a feeling of inadequacy, but also how peer-to-peer teaching can create a safe environment which inspires learning [6, 10, 33, 34].

### The longitudinal impact for the SFs

Denmark has adapted the CanMeds seven roles of a physician model, which describes the foundation of the necessary competences of a physician [35]. The SFs expressed that they developed themselves in six of the seven roles of a physician as peer teachers on this course, namely: medical expert, communicator, collaborator, leader, scholar and professional.

The medical expert role is developed through teaching. In the patient safety course topics such as the ABCDE assessment and Basic Life Support are used to teach the course participants about non-technical skills. The SFs expressed that their own skills within these areas improved as they trained hundreds of medical students during their work in the patient safety course. The SFs have expanded their knowledge of patient safety and can act as ambassadors of patient safety in the medical school.

To debrief medical students after a simulation is a complex matter, which developed the SF’s communicator role. Being able to listen intensely to every word, whilst observing body language and understanding the “non-verbal elements” of a dialogue takes practice, training and education. Furthermore, all medical students are different, and to teach these different personalities helps develop the SF’s ability to see, listen to, understand and respects their fellow human beings.; a key part of the communicator role. A previous study on long-term reactions to being a simulation facilitator also pointed out that the interaction with different people during the courses is both a challenge and a chance to develop [36].

As an SF you must take responsibility. You must be able to maintain an overview of both the simulation and the debriefing, and this developed their leadership role. “To teach a class is much like being the conductor (in a symphony), it is the broad lines, it is… everything” one of the SF said in the interview. This citation summarizes how teaching requires leadership and professionalism. As the SFs have the main role in teaching and managing the course, it is not only the personal leadership skills which develop, but also the development as an organizer. The SFs were managing the work schedule: they met and prepared in the morning before the course began, they were running the whole course-day, and they were undertaking the verbal evaluation after each course. Once every semester, the SFs met with faculty to develop the course based on evaluations from the course participants. These responsibilities that the SFs had forced them to develop as leaders, on both a personal level and on an organizational level.

We found that the SFs spent a lot of time educating each other within the SF team and the teacher role is developed through this. They endeavored to do the best for the course participants. Therefore, the SFs were observing and giving feedback to each other to optimize the teaching experience. We believe that the SFs were developing the role of the teacher, as the role is defined as “to apply relevant training methods and commit themselves to creating a positive learning environment.”

Overall, the SFs involved in this study describe much broader gains than previously outlined in the literature. A recent study has indicated that the benefit of peer-assisted learning seems to be greater for the peer-teachers than the pear-learners [13]. The value for the SFs have focused on both theoretical and practical gains [10] and preparing the medical students for their role as teachers during residency [37]. We speculate that our SFs are possibly better prepared for their future clinical work as a physician, as a result of this experience. Data on the longitudinal impact of being a SF is sparse but crucial to understanding if and how the SFs are better prepared. Our study has contributed to our understanding of their perceived gain. Future studies of their ability to use this in their role as a physician are needed.

### Discussion of methods and reflexivity

The response rate of the questionnaire was 92%, which implies that our results are representative of the course participants who attended the course. However, it is not necessarily representative for course participants participating over the years as the SFs might be more competent now than in the beginning.

The first author (JK) conducted the interviews with the SFs. JK was a 5^th^ year medical student at the time of the interviews, working as a SF and the coordinator of the peer teachers’ group. Hence, he knew the interviewed peer teachers prior to the interviews. This might have influenced the interviewed SFs to talk about subjects which will be favourable for JK and the study. To account for some of this bias, we explained to the participants that the interviews would be presented anonymously to the research group.

### Lessons learned

This study adds to our knowledge of peer-assisted learning in SBT using a patient safety course as a case. Although the content of the course was new for the SFs, we found that involving SFs in the development and running SBT was feasible. The SFs expressed that they developed them-selves within six of the seven CanMEd roles, which might help them overcome the gap from being a medical student to become a doctor.

## Conclusion

This study illustrates how medical students were involved in the co-development, delivery and implementation of a course in patient safety. The evaluation of the course shows that SFs managed to create a safe learning environment and that the course participants reacted positively to the learning objectives of the course. The SFs had different motivations for teaching and different perceptions of being a teacher. They express a positive feeling of being able to establish a safe learning environment, but also a feeling of inadequacy when teaching peers. In addition, the SFs expressed that they developed themselves both professionally and personally.

## Data Availability

Data are stored under secure conditions and are available from the corresponding author upon reasonable request.
